# Natural nitration of CXCL12 reduces its signaling capacity and chemotactic activity *in vitro* and abrogates intra-articular lymphocyte recruitment *in vivo*

**DOI:** 10.18632/oncotarget.11516

**Published:** 2016-08-23

**Authors:** Rik Janssens, Anneleen Mortier, Daiane Boff, Vincent Vanheule, Mieke Gouwy, Charlotte Franck, Olav Larsen, Mette M. Rosenkilde, Jo Van Damme, Flávio A. Amaral, Mauro M. Teixeira, Sofie Struyf, Paul Proost

**Affiliations:** ^1^ Laboratory of Molecular Immunology, Department of Microbiology and Immunology, Rega Institute, KU Leuven, B-3000 Leuven, Belgium; ^2^ Departamento de Bioquímica e Imunologia, Instituto de Ciências Biológicas, Universidade Federal de Minas Gerais, Belo Horizonte, Brasil; ^3^ Laboratory for Molecular Pharmacology, Department of Neuroscience and Pharmacology, Faculty of Health and Medical Sciences, The Panum Institute, University of Copenhagen, DK-2200 Copenhagen, Denmark

**Keywords:** chemokine, posttranslational modification, lymphocyte migration, extravasation, chemotaxis

## Abstract

The chemokine CXCL12/stromal cell-derived factor-1 is important for leukocyte migration to lymphoid organs and inflamed tissues and stimulates tumor development. *In vitro,* CXCL12 activity through CXCR4 is abolished by proteolytic processing. However, limited information is available on *in vivo* effects of posttranslationally modified CXCL12. Natural CXCL12 was purified from the coculture supernatant of stromal cells stimulated with leukocytes and inflammatory agents. In this conditioned medium, CXCL12 with a nitration on Tyr^7^, designated [3-NT^7^]CXCL12, was discovered via Edman degradation. CXCL12 and [3-NT^7^]CXCL12 were chemically synthesized to evaluate the biological effects of this modification. [3-NT^7^]CXCL12 recruited β-arrestin 2 and phosphorylated the Akt kinase similar to CXCL12 in receptor-transfected cells. Also the affinity of CXCL12 and [3-NT^7^]CXCL12 for glycosaminoglycans, the G protein-coupled chemokine receptor CXCR4 and the atypical chemokine receptor ACKR3 were comparable. However, [3-NT^7^]CXCL12 showed a reduced ability to enhance intracellular calcium concentrations, to generate inositol triphosphate, to phosphorylate ERK1/2 and to induce monocyte and lymphocyte chemotaxis *in vitro*. Moreover, nitrated CXCL12 failed to induce *in vivo* extravasation of lymphocytes to the joint. In summary, nitration on Tyr^7^ under inflammatory conditions is a novel natural posttranslational regulatory mechanism of CXCL12 which may downregulate the CXCR4-mediated inflammatory and tumor-promoting activities of CXCL12.

## INTRODUCTION

The chemokine CXCL12 is a member of the CXC subgroup of chemotactic cytokines, which was previously purified from cell supernatant of the murine bone marrow stromal cell line MS-5 (hence its functional name stromal cell-derived factor-1/SDF-1) [1]. In addition to peripheral blood mononuclear cells (PBMCs), CXCL12 attracts neutrophils and CD34^+^ progenitor cells [2–5]. Recently, neutrophil-derived CXCL12-containing vesicles have been reported to be crucial for subsequent T cell migration to influenza-infected lungs [6]. CXCL12 also proved to be a co-stimulator for T cells [7]. Furthermore, CXCL12 promotes the adhesion of lymphocytes to activated endothelial cells and the subsequent transendothelial migration of these leukocytes [8–11]. CXCL12 fulfills this function in both an inflammatory context and during homeostatic trafficking of lymphocytes [12, 13]. CXCL12 controls circulating neutrophil levels and aids in the rapid release of neutrophils from the bone marrow during inflammation [14]. CXCL12 has important homeostatic functions in B cell lymphopoiesis and is responsible for the homing of hematopoietic stem and progenitor cells to the bone marrow [15–20]. In contrast to other chemokines, CXCL12 is vital for embryogenesis, since CXCL12 knock-out mice are non-viable animals which die *in utero* or shortly after birth [16, 21]. This chemokine is responsible for the trafficking of endothelial progenitors to peripheral tissues to assist in angiogenesis, wound healing and tissue repair [22–24]. By using its angiogenic and migration-inducing properties, different types of cancer cells exploit the CXCL12/CXCR4 axis to promote tumoral angiogenesis, growth and metastasis [25–31]. Furthermore, CXCL12 plays a role in several autoimmune diseases like rheumatoid arthritis (RA) and multiple sclerosis (MS) [32–34].

CXCL12 binds the HIV co-receptor LESTR/fusin and acts as a natural competitor for HIV-1 entry in T cells [35]. This receptor, now known as CXCR4, is the G protein-coupled receptor (GPCR) for CXCL12. CXCR4 activation results in intracellular release of Ca^2+^ and subsequent cellular responses like chemotaxis [29]. Moreover, CXCR7 was identified as a seven transmembrane-spanning receptor for both CXCL11 and CXCL12 [36, 37]. Since this receptor does not signal through G proteins but can activate β-arrestin-dependent signaling pathways, it was recently renamed atypical chemokine receptor 3 (ACKR3) [38, 39]. ACKR3 has a scavenging function, removing CXCL12 from the environment [40]. In addition to seven transmembrane-spanning receptors, CXCL12 also interacts with glycosaminoglycans (GAGs) such as heparin and heparan sulfate [41].

Chemokines are regulated at multiple levels to control inflammation and homeostasis. Inflammatory chemokine expression can be upregulated by local transcription and stabilization of unstable chemokine mRNA following inflammatory stimuli [42]. They can also be stored in Weibel-Palade bodies or other secretory storage granules in endothelial cells, ready to be released upon an inflammatory stimulus [43]. Another regulation mechanism of chemokines, in particular CXCL12, occurs through alternative splicing at the translational level. CXCL12 is known to have six human splice variants (CXCL12 α to φ) with different forms being expressed in different tissues [44–46]. Furthermore, regulation of chemokine activity has also been reported to occur via posttranslational modification [47, 48]. These alterations include degradation, truncation at the N- or C-terminus and citrullination [47, 48]. The non-degrading modifications can either inactivate, reduce or enhance the chemokine function [49–52]. To become biologically active *in vivo*, CXCL5 even needs to be N-terminally truncated [53]. Since CXCL12 is a homeostatic chemokine and is important in many inflammatory processes, posttranslational regulation is necessary to control the levels of active chemokine [54]. Carboxypeptidase N and M, present in the blood circulation and membrane-bound to bone marrow cells respectively, remove the C-terminal lysine of CXCL12α, reducing its biological activity [55, 56]. Also N-terminal truncation reduces the biological activity of CXCL12 and occurs in blood by the activity of CD26 [dipeptidyl peptidase IV (DPPIV)], cathepsin G, different members of the MMP family and neutrophil elastase [54, 57–62]. Recently, N-terminally truncated variants of CXCL12 were purified from human plasma, confirming the *in vivo* processing of CXCL12 by some of the enzymes listed above [63]. Also, *in vitro* citrullination of Arg^8^ reduces the biological function of CXCL12, whereas citrullination of Arg^8^, Arg^12^ and Arg^20^ or citrullination of all five arginine residues completely abolishes CXCL12 activity on CXCR4 [64].

Since natural CXCL12 is highly produced by bone marrow stromal cells, our interest was to analyze how this chemokine would be secreted and posttranslationally modified under inflammatory conditions. For this reason a stromal cell line was cocultered with leukocytes and stimulated with inflammatory agents. In addition to previously reported truncated CXCL12 forms, we here report the identification and functional characterization *in vitro* and *in vivo* of a novel natural posttranslationally modified CXCL12 form with nitration on Tyr^7^.

## RESULTS

### Production and purification of nitrated CXCL12 from conditioned media of stimulated bone marrow stromal cells

**Figure 1:**
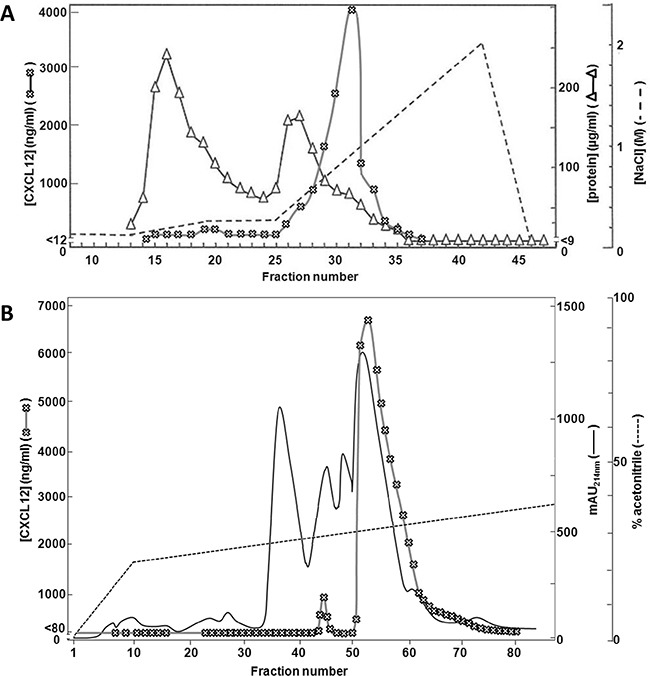
Purification of CXCL12 from stromal cell conditioned medium MS-5 cells were stimulated with IFN-γ and the double-stranded RNA poly I:C in the presence of neutrophils and PBMCs. 72h after stimulation, conditioned medium was collected and purified using two consecutive chromatographic steps. Panel **A** shows the results from the heparin-Sepharose affinity chromatography. Proteins were eluted using a two-step sodium chloride gradient (dashed line). Total protein content was determined for each fraction using a BCA assay (open triangles). Total CXCL12 concentrations were determined for each fraction by ELISA (open crosses). Panel **B** shows the purification of the selected heparin-Sepharose fractions (i.e. fraction 29 to 32) by C8 RP-HPLC. Proteins were eluted using a gradient of acetonitrile in 0.1% (v/v) TFA (dashed line) and detected by their UV absorption (λ = 214nm, full line). CXCL12 concentrations were determined by ELISA (open crosses). mAU = milliabsorption units.

Bone marrow stromal cells were cocultered with primary leukocytes and stimulated with both the cytokine interferon-γ (IFN-γ) and the Toll-like receptor 3 ligand polyinosinic:polycytidylic acid (poly I:C). CXCL12 was purified from the conditioned media by heparin affinity chromatography and reversed phase (RP) chromatography (Figure 1). The first purification step was based on the ability of the proteins to bind negatively charged GAGs (Figure 1A). The bulk amount of proteins either did not bind to the column or eluted early (at <0.5M) in the NaCl gradient, as evidenced by the high total protein concentrations in the first 28 column fractions. As determined by a specific CXCL12 ELISA, CXCL12 eluted later (>0.5M) in the gradient (fractions 29 to 36). The heparin affinity chromatography fractions containing the highest concentrations (>1000ng/ml) of CXCL12 were pooled as input for the RP-HPLC purification with on-line UV monitoring (Figure 1B). As detected by a specific CXCL12 ELISA, a single peak of CXCL12 eluted at 30% acetonitrile. After analysis by SDS-PAGE, which confirmed the presence of pure CXCL12 protein in the fractions (data not shown), the sequence of the purified CXCL12 was determined using Edman degradation. Interestingly, N-terminal peptide sequencing showed, in addition to the well-studied CXCL12(3-68) [61, 62], the presence of nitro-tyrosine on position 7 of CXCL12, referred to as [3-NT^7^]CXCL12. No unmodified tyrosine was detected by Edman degradation on CXCL12 eluting in fractions 52, 60 and 68 covering the elution peak of natural CXCL12. However, the nitrated form of CXCL12 was not produced by unstimulated stromal cells (without addition of leukocytes or inflammatory stimuli).

### Production of synthetic nitrated CXCL12 variants

**Figure 2:**
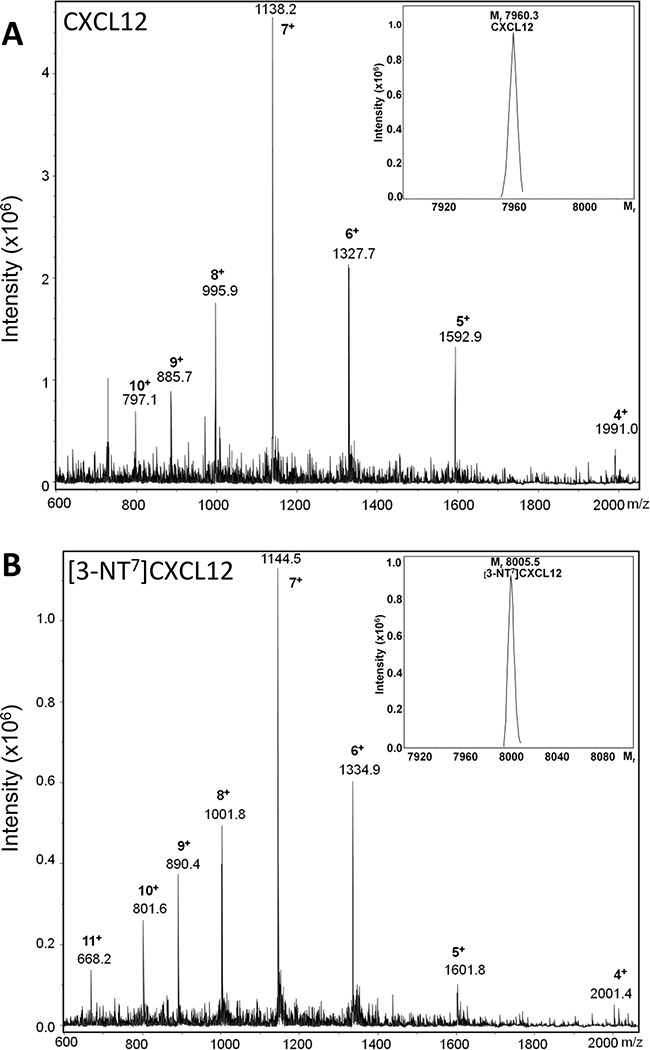
Mass spectrometric analysis of synthetic CXCL12 and [3-NT^7^]CXCL12 Chemically synthetized human CXCL12 and [3-NT^7^]CXCL12 were deprotected, folded and subsequently purified using RP-HPLC. Fractions were selected based on purity and correct relative molecular mass (M_r_). Shown are the averaged mass spectrum of the final pool of synthetic purified and folded CXCL12 (panel **A**) and [3-NT^7^]CXCL12 (panel **B**) with the number of charges for the detected ions, ion intensities and corresponding mass to charge ratios (m/z) for the multiply charged ions. The inserts in panels A and B show the deconvoluted mass spectra with the M_r_ of the uncharged proteins calculated using the Bruker deconvolution software.

To investigate the *in vitro* and *in vivo* biological effects of N-terminal nitration of CXCL12, we first attempted to chemically nitrate CXCL12 using peroxynitrite according to a previously described procedure [65]. However, following this protocol, i.e. an incubation of chemokine with 1mM of peroxynitrite in 100μl of PBS with 0.1% (w/v) BSA for 15 minutes at 37°C, no intact nitrated chemokine was detected (data not shown). Indeed, as the chemical treatment of CXCL12 was performed during different incubation periods, we detected more advanced degradation of the chemokine as incubation times were longer. As such, no intact chemokine was detected by SDS-PAGE after an incubation period longer than 8 minutes (data not shown). Because incubation of CXCL12 with peroxynitrite resulted in rapid CXCL12 degradation, we prefered to produce the nitrated CXCL12 synthetically, incorporating a nitrotyrosine at position 7, as was detected by Edman degradation on natural CXCL12. Both intact human CXCL12 and [3-NT^7^]CXCL12 were chemically synthesized by Fmoc solid phase peptide synthesis, folded and purified. The M_r_ of each produced chemokine was confirmed by ion trap mass spectrometry (Figure 2). Unmodified synthetic CXCL12 and [3-NT^7^]CXCL12 had an experimental M_r_ of 7960.3 and 8005.5, respectively (theoretical M_r_ is 7959.4 and 8004.4, respectively). The differences in M_r_ of the two CXCL12 forms with their theoretical M_r_ are well within the accuracy of the used mass spectrometer.

### Glycosaminoglycan and CXCR4 binding properties of [3-NT^7^]CXCL12 equal those of native CXCL12

**Figure 3:**
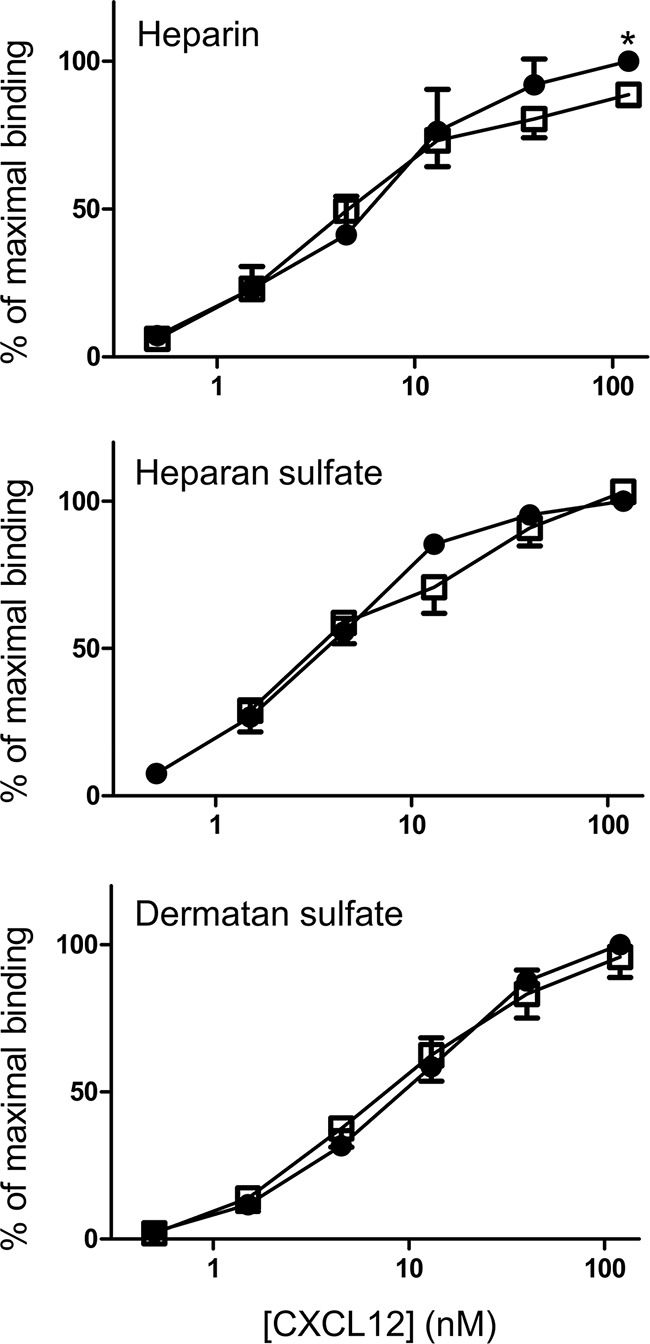
Binding properties of CXCL12 and its nitrated form to heparin, heparan sulfate and dermatan sulfate Binding of CXCL12 (•, filled circles) and [3-NT^7^]CXCL12 (□, open squares) to heparin, heparan sulfate and dermatan sulfate are shown. CXCL12 and [3-NT^7^]CXCL12 (ranging from 0.5 nM to 120 nM in a threefold serial dilution) were added to GAG-coated wells. The amount of bound chemokine was determined using biotinylated anti-human CXCL12 antibodies and is represented by the mean percentage (n=6; ± SEM) of the absorbance at 450nm measured with 120nM CXCL12. Statistical comparison between CXCL12 and [3-NT^7^]CXCL12 was performed using the Mann-Whitney *U* test (* p < 0.05).

It was tested whether the presence of a nitrotyrosine in the N-terminal region of CXCL12 altered its capacity to bind GAGs. Figure 3 shows the dose response curves for binding of CXCL12 to heparin, heparan sulfate and dermatan sulfate. The absorbance of the highest dose (i.e. 120nM) of native, unmodified CXCL12 was set to 100% for each experiment. CXCL12 and [3-NT^7^]CXCL12 binding to each GAG was calculated as the percentage of the signal from 120nM CXCL12 in each experiment. The figure shows dose response curves with similar slopes for both CXCL12 forms. Only at the highest dose of 120nM a minor but significant reduction in binding to heparin could be detected for [3-NT^7^]CXCL12 compared to CXCL12.

**Figure 4:**
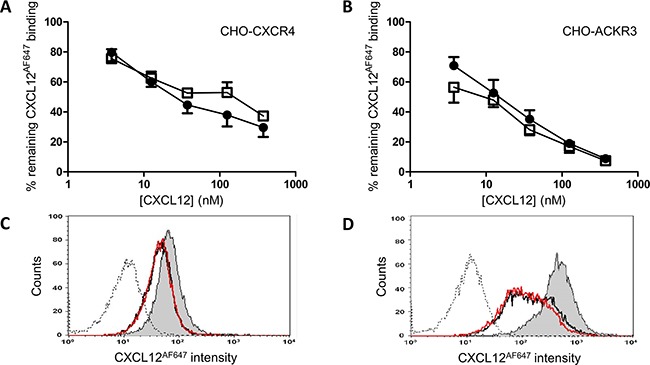
Binding properties of CXCL12 and [3-NT^7^]CXCL12 to CXCR4 and ACKR3 CXCL12 (•, filled circles) and [3-NT^7^]CXCL12 (□, open squares) were added (concentrations ranging from 3.75nM to 375nM) to CHO-CXCR4 cells (panel **A**) or CHO- ACKR3 cells (panel **B**), together with 12.5nM CXCL12^AF647^ while the cells were kept on ice during the one hour incubation period. The results shown are the mean percentages (±SEM) of fluorescence compared to the control where only CXCL12^AF647^ was added to the cells (n=8 for the CXCR4 experiments, n=4 for ACKR3). Statistical comparison between CXCL12 and [3-NT^7^]CXCL12 was performed using the Mann-Whitney *U* test. Panels **C** and **D** show FACS data from a representative experiment with CHO-CXCR4 and CHO-ACKR3, respectively. The remaining fluorescence of CXCL12^AF647^ after competition with 37.5nM CXCL12 (black line) or 37.5nM [3-NT^7^]CXCL12 (red line) are represented by two almost completely overlaying curves. The histogram where only CXCL12^AF647^ was added to the cell (grey area) shows the maximal fluorescence signal. The unstained control is also shown (dotted line).

In addition to binding to GAGs, binding of the native and nitrated CXCL12 to the CXCL12 receptors CXCR4 and ACKR3 was also evaluated in CXCR4-transfected and ACKR3-transfected CHO cells (Figure 4A and 4B, respectively). The ability of CXCL12 and [3-NT^7^]CXCL12 to compete with 12.5nM of Alexa Fluor 647 labeled CXCL12 (CXCL12^AF647^) was comparable for both receptors. In Figure 4C and 4D, this comparable binding to both receptors was shown as raw FACS data for a representative experiment.

### CXCR4 internalization by [3-NT^7^]CXCL12 is moderately reduced compared to CXCL12

**Figure 5:**
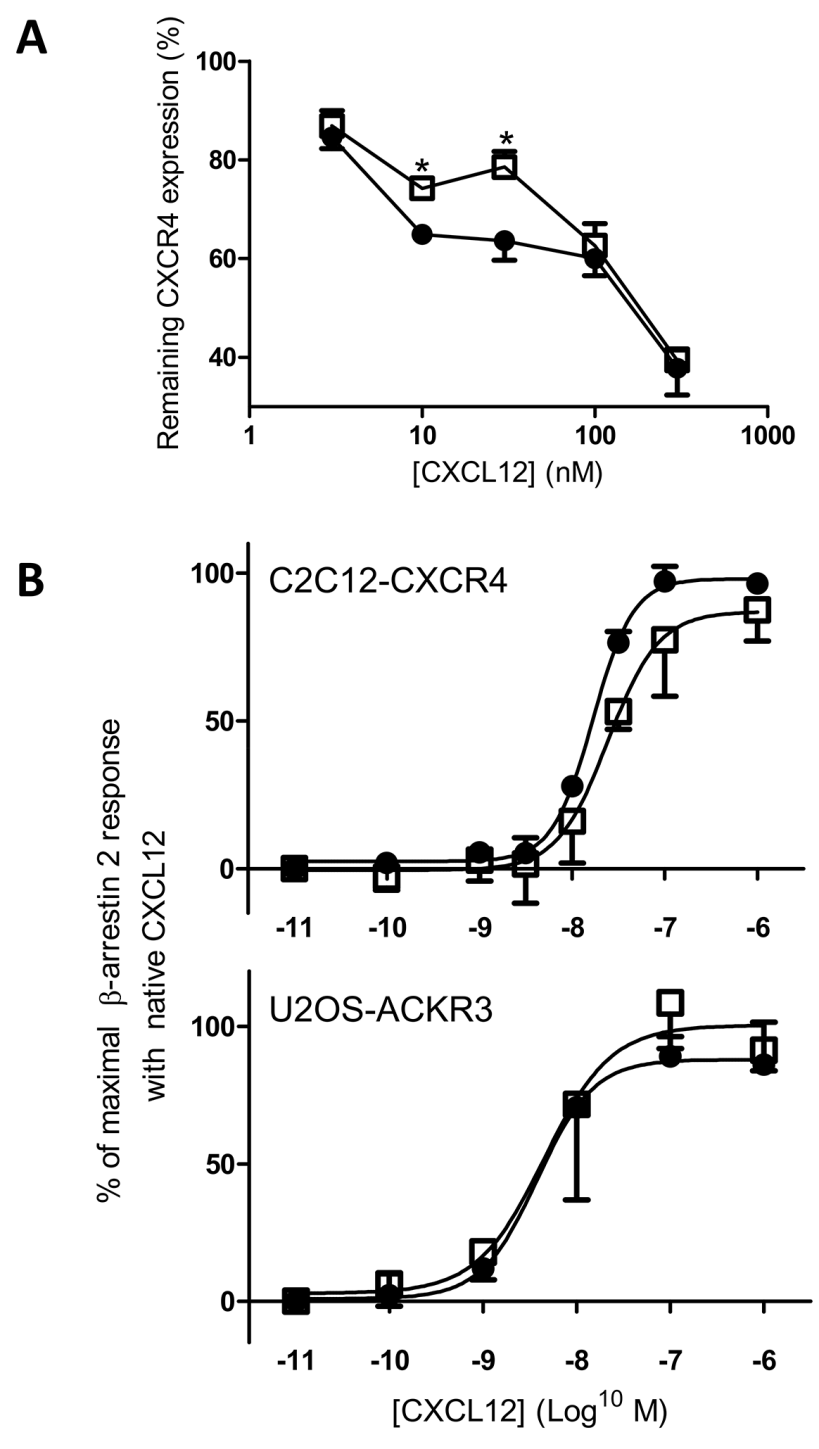
CXCR4 internalization and β-arrestin 2 recruitment through CXCR4 and ACKR3 in response to CXCL12 and [3-NT^7^]CXCL12 stimulation CXCL12 and its nitrated counterpart were compared for their ability to induce CXCR4 internalization (panel A) and β-arrestin 2 recruitment (panel B) through activation of CXCR4 or ACKR3. **A.** Using specific anti-CXCR4 antibodies, remaining receptor expression on CHO-CXCR4 cells was tested after one hour incubation at 37°C with CXCL12 (•, filled circles) or [3-NT^7^]CXCL12 (□, open squares). Both forms were added at concentrations ranging from 3.75 to 375nM. For each experiment, the fluorescence measured after stimulation with buffer only was set to 100%. Statistical analysis (n=4; ± SEM) was performed using the Mann-Whitney *U* test (* p < 0.05). **B.** The recruitment of β-arrestin 2 in function of the ligand concentration (0.01nM to 1μM) was compared for CXCL12 (•, filled circles) and [3-NT^7^]CXCL12 (□, open squares) in CXCR4-transfected C2C12 cells (n ≥ 4) or ACKR3-transfected U2OS cells (n=3). The data shown are a mean percentage (± SEM) of the maximal β-arrestin 2 recruitment with unmodified CXCL12. Statistical differences between the two CXCL12 forms were analyzed using the Mann-Whitney *U* test.

Receptor internalization was also investigated in addition to binding characteristics. In general, the N-terminal part of chemokines is important for receptor activation. Figure 5A shows the dose-dependent capacity of CXCL12 and [3-NT^7^]CXCL12 to induce CXCR4 internalization in CXCR4-transfected CHO cells. At high or low concentrations both forms were equally potent. However, at moderate concentrations (12.5nM and 30nM) intact CXCL12 was significantly better at inducing CXCR4 internalization compared to its nitrated form. Previous reports showed that CXCR4 internalization is mediated by β-arrestin 2 recruitment to the cell membrane [66]. Therefore, we also tested whether CXCL12 and [3-NT^7^]CXCL12 display a different potency to induce β-arrestin 2 recruitment to CXCR4 or ACKR3 (Figure 5B). In this assay, although there was a trend towards reduced β-arrestin 2 recruitment to CXCR4 with [3-NT^7^]CXCL12, we could not detect a significant difference between both forms. Also for ACKR3 we could not measure an altered β-arrestin 2 recruitment.

### Analyses of signal transduction capacities of [3-NT^7^]CXCL12 show a reduced calcium signaling potency compared to native CXCL12

**Figure 6:**
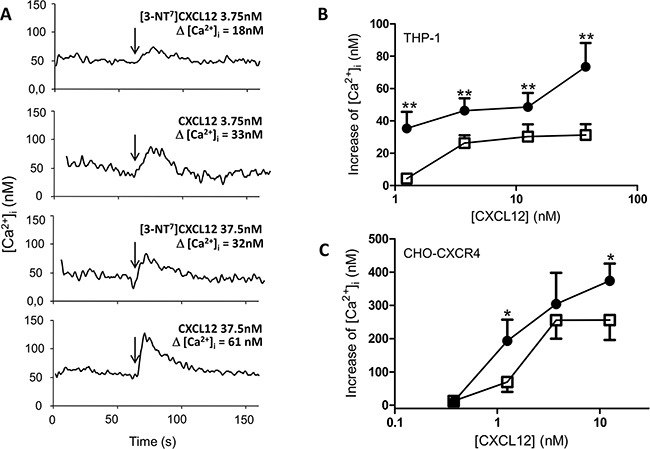
Calcium mobilization following CXCR4 activation with CXCL12 and [3-NT^7^]CXCL12 **A.** Real-time changes of intracellular calcium levels (Δ[Ca^2+^]_i_) are shown in function of time. After 60 seconds, indicated by the arrows, 3.75nM or 37.5nM of CXCL12 or [3-NT^7^]CXCL12 are added to CHO-CXCR4 cells. The summarizing figures of these calcium mobilization experiments are shown in panel B and C. **B, C.** The increase of free intracellular calcium ions after stimulation of THP-1 cells (panel B; n= 6; ± SEM) or CHO-CXCR4 cells (panel C; n=7; ± SEM) with CXCL12 (•, filled circles) and [3-NT^7^]CXCL12 (□, open squares) was monitored (concentrations ranging from 0.375nM to 37.5nM).

In addition, we investigated different signaling pathways known to be activated by CXCL12. The increase in intracellular calcium concentration ([Ca^2+^]_i_) following stimulation of CHO-CXCR4 and THP-1 cells with CXCL12 and [3-NT^7^]CXCL12 was measured (Figure 6). A representative experiment with THP-1 cells is shown in Figure 6A. On THP-1 cells (Figure 6B), every applied dose of CXCL12 induced a significantly higher increase of [Ca^2+^]_i_ compared to its nitrated counterpart. For CHO-CXCR4 cells (Figure 6C), the response after stimulation of the cells with 1.25nM and 12.5nM of CXCL12 was significantly higher than the increase in [Ca^2+^]_i_ after stimulation of the cells with the same concentration of [3-NT^7^]CXCL12.

**Figure 7:**
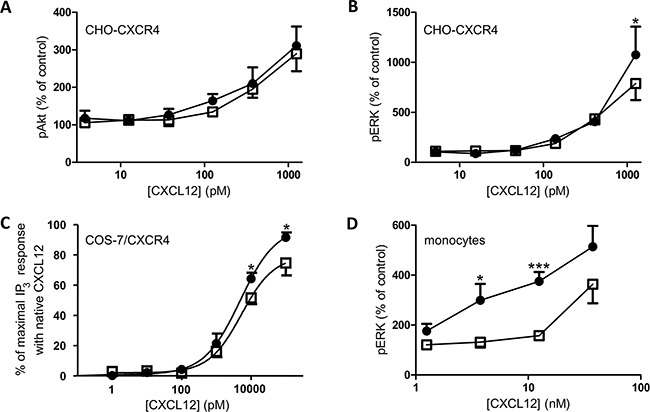
ERK, Akt and IP_3_ signal transduction pathways activated by CXCL12 and [3-NT^7^]CXCL12 The accumulation of second messengers after stimulation of different cell types with CXCL12 (•, filled circles) and [3-NT^7^]CXCL12 (□, open squares) is shown. **A, B.** The average accumulation (n=8; mean ± SEM) of phosphorylated Akt and ERK1/2 after a two minute stimulation of CHO-CXCR4 cells with the CXCL12 forms (concentrations ranging from 3.75pM to 1.25nM) was calculated as a percentage compared to vehicle stimulated cells. **C.** IP_3_ generation (n=6; mean ± SEM) after stimulation of CXCR4-transfected COS-7 cells with both CXCL12 forms at concentrations ranging from 1pM to 100nM was measured. **D.** Fresh monocytes were stimulated with the CXCL12 forms (concentrations ranging from 1.25nM to 37.5nM). The average accumulation (n=9; mean ± SEM) of phosphorylated ERK1/2 after a two minute stimulation was calculated as a percentage compared to vehicle stimulated cells. All statistical analyses for differences between both CXCL12 forms were performed using the Mann-Whitney *U* test (* p < 0.05, ** p < 0.01, *** p<0.001).

The phosphorylation of the kinases Akt and ERK after stimulation of CHO-CXCR4 cells with different concentrations of both CXCL12 forms was also investigated (Figure 7A and 7B). CXCL12 and [3-NT^7^]CXCL12 were equally potent in inducing Akt phosphorylation, whereas a significantly higher ERK1/2 phosphorylation was detected when the cells were stimulated with 1.25nM of CXCL12 compared to stimulation with 1.25nM [3-NT^7^]CXCL12. Evaluation of the formation of IP_3_ (Figure 7C) showed a significantly higher IP_3_ accumulation in the cytoplasm after stimulation of CXCR4-transfected COS-7 cells with 10nM and 100nM CXCL12 when compared to the nitrated CXCL12 form at these concentrations. Also ERK1/2 phosphorylation in human peripheral blood monocytes in response to CXCL12 or [3-NT^7^]CXCL12 was compared and a significantly higher phosphorylation after stimulation with 3.75nM and 12.5nM CXCL12 was detected.

### Compared to native CXCL12, the chemotactic potency of [3-NT^7^]CXCL12 for monocytic THP-1 cells as well as lymphocytes is reduced

**Figure 8:**
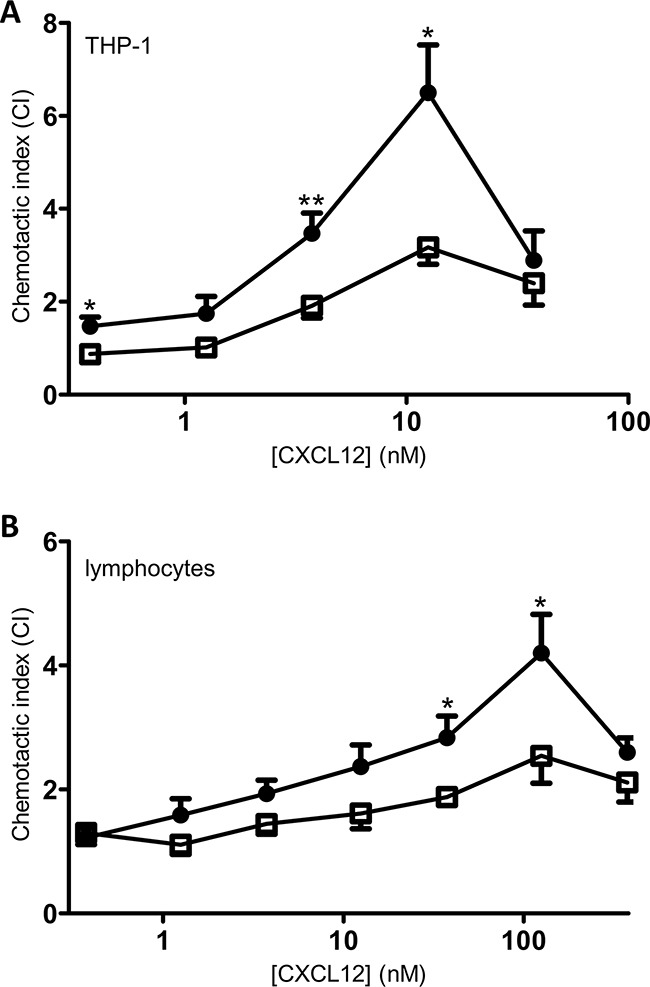
*In vitro* chemotaxis of monocytes and lymphocytes towards CXCL12 and [3-NT^7^]CXCL12 Migration of monocytic THP-1 cells (panel **A**; n=7) and freshly isolated PBMCs (panel **B**; n=9) was compared towards CXCL12 (•, filled circles) and [3-NT^7^]CXCL12 (□, open squares) and is represented by chemotactic indices (mean ± SEM). Statistical differences between the two CXCL12 forms were determined using the Mann-Whitney *U* test (* p < 0.05, ** p < 0.01).

A main activity of chemokines when added to the appropriate cells is the induction of a chemotactic response. Therefore, chemotaxis assays were performed using monocytic THP-1 cells and freshly isolated PBMCs as a source of lymphocytes. THP-1 cell chemotaxis assays showed that CXCL12 was able to induce significant monocyte chemotaxis as compared to buffer solution at all concentrations except 1.25nM (Figure 8A). Stimulation with 3.75nM or higher concentrations of [3-NT^7^]CXCL12 induced a significant chemotactic response. However, at 3.75nM and 12.5nM, native CXCL12 was significantly more potent at inducing chemotaxis when compared to its nitrated form (maximum chemotactic index of 6.5 ± 1 for CXCL12, compared to 3.2 ± 0.4 for [3-NT^7^]CXCL12).

Chemotaxis of freshly isolated lymphocytes through fibronectin-coated membranes after 4h of incubation in response to different concentrations of CXCL12 forms showed a similar bell-shaped dose response curve. Both forms were able to induce significant *in vitro* lymphocyte chemotaxis compared to buffer solution at any given dose above 1nM (Figure 8B). At 37.5nM and 125nM, native CXCL12 induced a significantly higher lymphocyte chemotactic response compared to nitrated CXCL12 (maximum CI of 4.2 ± 0.6 for CXCL12, compared to 2.5 ± 0.4 for [3-NT^7^]CXCL12).

### [3-NT^7^]CXCL12 has a strongly reduced potency to attract lymphocytes *in vivo*

**Figure 9:**
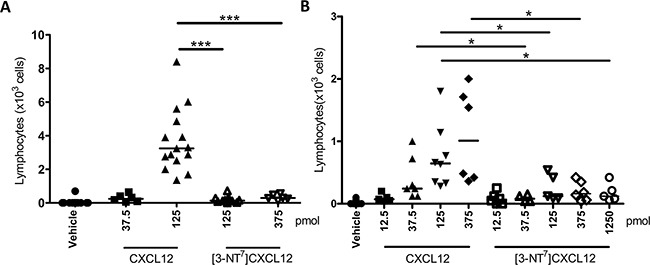
*In vivo* recruitment of lymphocytes into the joint towards CXCL12 and [3-NT^7^]CXCL12 Vehicle, CXCL12 or [3-NT^7^]CXCL12 were injected in a final volume of 10μl into the tibiofemoral articulation of C57BL/6 mice treated with sitagliptin. 3h post injection, the number of leukocytes migrated into the joint was determined and the % lymphocytes was counted differentially on May-Grünwald-Giemsa stained cytospins. Each symbol represents an individual mouse (n ≥ 5). Horizontal lines indicate the median number of lymphocytes for each treatment group. The experiments were performed independently in Brazil **A.** and Belgium **B.** Statistical differences between groups of mice were determined using the Mann-Whitney *U* test (* p < 0.05, *** p < 0.001). All CXCL12 and [3-NT^7^]CXCL12 doses except 12.5pmol [3-NT^7^]CXCL12 were significantly different from vehicle injection (p < 0.01).

Given the reduced potency of [3-NT^7^]CXCL12 to induce chemotaxis of monocytes and lymphocytes *in vitro*, the ability of [3-NT^7^]CXCL12 to induce extravasation of lymphocytes was investigated *in vivo*. We evaluated CXCL12-induced lymphocyte recruitment into the joint upon injection of the chemokine in the tibiofemoral articulation. This model has the advantage of a very low basal leukocyte count upon vehicle injection. All tested chemokine doses of CXCL12 or [3-NT^7^]CXCL12 depicted in Figure 9A caused significant extravasation of lymphocytes as compared to vehicle injection. However, an injection of 125pmol of CXCL12 caused significantly higher lymphocyte extravasation when compared to an injection of the same dose of [3-NT^7^]CXCL12 (3.3 × 10^3^ lymphocytes vs. 0.16 x10^3^ lymphocytes). Even an injection with 375pmol of nitrated CXCL12 only resulted in a few lymphocytes migrating into the joint (median amount of 0.3 × 10^3^ cells). Additional experiments were performed in a different animalium using a wider range of concentrations of the CXCL12 forms (Figure 9B). Here, only 12.5pmol of [3-NT^7^]CXCL12 was not able to recruit a significant amount of lymphocytes compared to vehicle. At other doses, [3-NT^7^]CXCL12 induced a statistically significant but limited infiltration of lymphocytes in the joint. At doses of 37.5pmol, 125pmol and 375pmol CXCL12 was significantly better at recruiting lymphocytes compared to [3-NT^7^]CXCL12.

## DISCUSSION

Reactive nitrogen species, of which nitric oxide (NO) is characterized the best, are a key part of the immune system and can have both a beneficial or malignant role [67,68]. NO is a short-lived free radical that can freely diffuse across cell membranes to fulfill its functions [67]. The production of NO is based on the balance between three enzymes that use L-arginine as a substrate, i.e. nitric oxide synthase (NOS) and arginases 1 and 2. The arginases take part in the urea cycle where the end products are ornithine and urea, whereas NOS produces nitric oxide and superoxide anion [68]. The NOS family comprises three mostly constitutive NOS enzymes: neuronal (nNOS), endothelial (eNOS) and mitochondrial (mtNOS). In addition, a fourth member, inducible NOS (iNOS), is controlled by different cytokines and microbial triggers that either increase or decrease its expression, depending on the use of different signaling pathways [67]. Important NO producing cells are macrophages, assisting in pathogen removal, anti-tumor activity and tissue necrosis [69–71]. However, also immunosuppressive effects and the up- and downregulation of several cytokines, chemokines and growth factors have been described, adding to the complexity of NO in the immune system [67]. Also neutrophils and eosinophils produce NO in their anti-microbial and anti-tumoral function [72, 73]. Under sustained inflammation, reactive oxygen species (ROS) like superoxide anion reach high enough concentrations to either directly oxidize molecules or react rapidly with the produced NO to form the highly reactive peroxynitrite (ONOO^−^) that can nitrate tyrosine residues [74]. The presence of nitro-tyrosine is a commonly used marker of inflammation, since it is the result of the presence of both ROS and RNS in the microenvironment [74–77].

Many proteins can be subjected to tyrosine nitration. The effects of this posttranslational modification are diverse and may result in a decrease or loss of function, no alteration in activity or an increase of protein activity [68, 75, 77]. For chemokines, only the *in vitro* nitration of CCL2, CCL5 and CXCL12 by peroxynitrite have been described [78]. Chemical nitration of CCL5 caused a significant reduction in chemotactic activity for eosinophils [79]. However, the reported data on CCL2 are less conclusive. One report shows that the treatment of CCL2 with peroxynitrite resulted in an impaired capacity to attract antigen-specific CD8^+^ T cells into the tumor tissue in mice, whereas CD14^+^ monocyte chemotaxis remained unaltered [65]. Sato *et al*., however, observed a reduced monocyte chemotactic activity after CCL2 nitration [80]. Also CXCL12 was reported to be nitrated chemically, which resulted in a loss of CD8^+^ T cell attraction [65]. However, in our hands the incubation of CXCL12 with peroxynitrite under the published conditions resulted in destruction of the protein. As discussed before, CXCL12 fulfills several functions in inflammation, and is often linked with enhanced inflammation. However, CXCL12 also functions as a protective chemokine in other conditions. In experimental autoimmune encephalitis (EAE), for example, CXCL12 has been reported to have a protective role, attracting and redirecting the polarization of T helper cells to regulatory, IL-10-producing T cells [81]. As mentioned before, in an inflammatory environment often ROS and RNS are coproduced. Recently, it was reported that NO production in the inflamed central nervous system (CNS) inhibits the expression of CXCL12, preventing it to fulfill its protective function [82]. However, it is not known how CXCL12 is posttranslationally modified in an inflamed environment.

In our study, MS-5 bone marrow stromal cells were cocultured with neutrophils and PBMCs with the addition of the inflammatory stimuli IFN-γ and poly I:C. After purification of the conditioned medium, we identified for the first time naturally nitrated CXCL12: [3-NT^7^]CXCL12. These data confirm that CXCL12 can be subjected to tyrosine nitration and, moreover, that this modification can occur in a cell environment without CXCL12 degradation.

Using synthetic CXCL12 forms, the biological effect of CXCL12 nitration was verified in several assays. Binding to GAGs is indispensable for CXCL12 and chemokine activity in general, as it is important for the formation of a chemokine gradient, promotes oligomerization and protects the chemokine from CD26-mediated cleavage in blood and tissues [41, 83, 84]. The GAG binding experiments did not show a significant difference between CXCL12 and [3-NT^7^]CXCL12 for heparin, heparan sulfate and dermatan sulfate. Previous reports stated that the *BBXB* domain, where *B* stands for a basic amino acid and *X* for any other non-basic amino acid, is a key component in chemokine-GAG interaction [85]. As the tyrosine at position 7 is not located in the vicinity of these domains, a difference in GAG binding due to its modification is unlikely. Also, the binding to both CXCR4 and ACKR3 was unaltered. Internalization and activation of CXCR4, considering β-arrestin 2, Akt and ERK1/2, were not or only moderately altered in the used cell lines. Also IP_3_ signaling was moderately but significantly reduced. However, ERK 1/2 phosphorylation was significantly reduced in fresh monocytes. Also β-arrestin 2 recruitment following ACKR3 activation was not affected by tyrosine nitration. This is in agreement with the fact that Tyr^7^ predominantly has a structural role and is not a key factor for receptor binding and activation, as was reported by Crump *et al.* [86].

Clear effects of Tyr^7^ nitration were seen in calcium mobilization assays. CXCL12 was confirmed to be able to induce the mobilization of calcium as a second messenger [87]. Nitration of the N-terminal tyrosine caused a significant reduction in the ability of CXCL12 to induce calcium fluxes in the cytoplasm. The clear effects of nitration on signal transduction through ERK1/2, IP_3_ and especially calcium following CXCR4 activation contrast with the absence of effect of nitration on the β-arrestin 2 recruitment and might indicate a bias towards specific signaling pathways through CXCR4.

Signal transduction following the binding of a chemokine to its receptor leads in most cases to a chemotactic response of the target cell [41, 88]. In this study, we show that N-terminal tyrosine nitration of CXCL12 reduces its potency to attract lymphocytes *in vitro*. In addition, we also show significant reduction in chemotactic activity on monocytic THP-1 cells. This reduced migratory response of lymphocytes was further highlighted by the strong effect of nitration of CXCL12 *in vivo*, where [3-NT^7^]CXCL12 was barely able to attract lymphocytes to the tibiofemural articulation. The stronger effect *in vivo* than *in vitro* could be the consequence of multiple factors that were affected by CXCL12 nitration.

In conclusion, we were the first to demonstrate that CXCL12 can be N-terminally nitrated in an inflamed environment. This posttranslational nitration did not have strong consequences on binding to GAGs and CXCR4 or ACKR3 *in vitro*. Although not influencing Akt signaling, we observed a significant reduction in the second messengers ERK, IP_3_ and calcium, which resulted in a reduced lymphocyte and monocytic THP-1 cell chemotaxis and was confirmed for lymphocytes in an *in vivo* setting. As such, nitration of CXCL12 may down-regulate the function of CXCL12 in a pathological context.

## MATERIALS AND METHODS

### Cells

The murine bone marrow stromal cell line MS-5 [89] was obtained from Prof. Y. Beguin (University of Liège, Liège, Belgium) [90] and cultured in Iscove's Modified Dulbecco's Medium (IMDM; Gibco, Auckland, New Zealand), enriched with 10% (v/v) fetal bovine serum (FBS; HyClone, Cramlington, UK), 5μg/ml gentamycin (Sigma-Aldrich, St. Louis, MO) and 3% (w/v) sodium bicarbonate (Gibco). Once confluent monolayers were obtained, 3% (v/v) FBS and 3% (w/v) sodium bicarbonate enriched IMDM was used.

The chinese hamster ovary (CHO) cell line transfected with CXCR4 was provided by Prof. Dr. M. Parmentier from the Institute of Interdisciplinary Research in Human and Molecular Biology (IRIBHM) at the Université Libre de Bruxelles, Brussels, Belgium. CHO cells were cultivated in Ham's F-12 growth medium (Lonza, Basel, Switzerland) including 10% (v/v) FBS, 400μg/ml G418 (Gibco) and 250μg/ml zeocin (Invitrogen, Carlsbad, CA). Human monocytic THP-1 cells (American Type Culture Collection, Manassas, VA) were cultured in Roswell Park Memorial Institute medium 1640 with glutamine supplement (RPMI 1640 - GlutaMAX; Gibco) which was further enriched with 10% (v/v) FBS and 3% (w/v) sodium bicarbonate. Both THP-1 and CHO cells were subcultivated two days prior to the signaling or cell migration assays.

The mouse myoblast cell line C2C12 transfected with CXCR4 was obtained from DiscoveRx (Fremont, CA) and was cultured in Dulbecco's Modified Eagle's Medium 1885 (DMEM; Gibco), containing 20% (v/v) FBS and penicillin, streptomycin and L-glutamine. COS-7 cells transfected with CXCR4 were cultivated in DMEM 1885 enriched with 10% (v/v) FBS and penicillin, streptomycin and L-glutamine. U2OS cells were cultured in Minimal Essential Medium Alpha (MEM-alpha; Gibco) supplemented with 10% (v/v) FBS and penicillin, streptomycin, hygromycin and L-glutamine. The COS-7 cells were cultured in an environment containing 10% CO_2_, all other cells were kept at the usual 5% CO_2_. Neutrophils, PBMCs and lymphocytes were isolated from buffy coats that were freshly provided by the Belgian Red Cross (Mechelen, Belgium) as previously described [91].

### ELISA

The detection and quantification of CXCL12 was performed through enzyme-linked immunosorbent assays (ELISAs) using specific antibodies as described by Loos *et al.* [92]. Briefly, total CXCL12 concentrations were measured by coating a 96-well plate (Costar, Corning, NY) with 2μg/ml monoclonal anti-human/anti-mouse CXCL12 (Clone 79014; R&D Systems, Minneapolis, MN) and detecting the bound antigen with polyclonal biotinylated anti-human CXCL12 (Peprotech, Rocky Hill, NJ) at 250ng/ml. After incubation with streptavidine-labeled horseradish peroxidase (R&D Systems), CXCL12 concentrations were determined using the enzymatic activity of the horseradish peroxidase. This enzyme oxidizes the colorless 3,3′,5,5′-tetramethylbenzidine (TMB; Sigma-Aldrich) substrate into a blue product, using 0.004% (v/v) H_2_O_2_ as oxidizing agent.

### Chemokine production, purification and identification

MS-5 cells were stimulated with interferon-γ (Peprotech) and the double-stranded RNA poly I:C (Sigma Aldrich) at final concentrations of 20ng/ml and 10μg/ml, respectively. Together with these inducers, also buffy coat-derived purified neutrophils and PBMCs were added at concentrations of, respectively, 3 × 10^5^ cells/ml and 4 × 10^5^ cells/ml [91]. Conditioned medium was harvested after 72 hours and stored at −20°C prior to purification.

A first purification step was performed using affinity chromatography with a heparin-Sepharose CL-6B column (1.6 × 40cm, 70ml volume; GE Healthcare, Uppsala, Sweden). The column was equilibrated with 50mM Tris-HCl and 50mM NaCl, pH 7.4, at a flow rate of 10ml/hour before the sample was loaded. After washing the column with equilibration buffer the sample was eluted with a multi-step NaCl gradient ranging from 0.2 M to 2 M in 50mM Tris-HCl, pH 7.4, at a flow rate of 20ml/hour. Fractions of 5ml were collected and stored at −20°C.

Chemokine concentrations were assessed in these fractions by ELISA, whereas purity and total protein concentration were determined using sodium dodecyl sulphate polyacrylamide gel electrophoresis (SDS-PAGE) on Tris/tricine gels under reducing conditions [93] and staining the protein with silver as described before [94]. Total protein concentrations were quantified by performing a bicinchoninic acid (BCA) assay on the collected fractions. Fractions containing chemokine were further purified by reversed-phase (RP)-HPLC using a C8 Aquapore RP-300 column (7μm, 220 × 2.1mm; PerkinElmer, Waltham, MA). This column was equilibrated with 0.1% (v/v) trifluoroacetic acid (TFA; Biosolve, Valkenswaard, The Netherlands) in ultrapure water. After loading the heparin-Sepharose fractions on the RP-HPLC column, proteins were eluted by a 0% to 80% acetonitrile gradient in 0.1% (v/v) TFA (Biosolve). Proteins were detected by UV (214nm) absorption and fractions of 0.4ml were collected and tested again using specific CXCL12 antibodies and SDS-PAGE with appropriate staining. N-terminal amino acid sequences of the proteins present in the fractions were determined by Edman degradation on a capillary protein sequencer (Procise 491 cLC, Applied Biosystems, Foster City, CA).

### Chemical synthesis of CXCL12 and its nitrated variant

Intact CXCL12 and its nitrated variant, [3-NT^7^]CXCL12, were chemically synthesized using fluorenyl methoxycarbonyl (Fmoc) chemistry on an Activo-P11 solid phase peptide synthesizer (Activotec, Cambridge, UK) as previously described [95], with the inclusion of UV monitoring and the possibility of a second coupling step. Briefly, at least two piperidine treatments (2 and 5 minutes) were used for every amino acid. As long as the UV signal of the removed Fmoc group remained higher than 25, additional piperidine treatments were performed with a maximum of 6 treatments (10, 15, 30 or 60 minutes incubation periods for the 3^rd^ up to 6^th^ incubation with piperidine, respectively). Performing more than 2 piperidine treatments triggers the double coupling module. In case of single coupling the added amino acid (10-fold excess compared to the amount of resin) was activated in 2-(1H-benzotriazol-1-yl)-1,1,3,3-tetramethyluronium hexafluorophosphate/hydroxybenzotriazole (HBTU/HOBt) (0.5M/0.5M) and coupled for 20 minutes. When a double coupling step was needed due to the difficult removal of the Fmoc group of the previous amino acid, the amino acid used for the second coupling was activated with 1-[bis(dimethylamino)methylene]-1H-1,2,3-triazolo[4,5-b]pyridinium 3-oxid hexafluorophosphate (HATU)/HOBt (0.5M/0.5M) and both the first and second coupling steps were elongated to one hour.

[3-NT^7^]CXCL12 has a nitro-group at the ortho-position of the benzene ring of the tyrosine residue at position 7. At this position, during the synthesis of [3-NT^7^]CXCL12, an Fmoc-protected 3-nitrotyrosine residue (Iris Biotech, Marktredwitz, Germany) was incorporated in the amino acid sequence instead of a regular Fmoc-tyrosine.

The synthetic CXCL12 products (CXCL12 and [3-NT^7^]CXCL12) were deprotected and cleaved from the resin by a 90 minute incubation at room temperature using a solution containing 0.75 g crystalline phenol, 0.5ml thioanisole, 0.25ml ethanedithiol and 0.5ml pure water in 10ml TFA. Hereafter, the resin was removed by filtration and the deprotected peptides were precipitated and washed using diethyl ether. After lyophilization, chemokines were dissolved in ultrapure water and loaded on a RP-HPLC Source 5RPC column (4.6 × 150mm; GE Healthcare). Elution of the chemokines was obtained in an acetonitrile gradient as described before [95]. A fraction (0.7%) of the eluent was split to an ion trap mass spectrometer (Bruker Daltonics, Bremen, Germany) using a flow splitter (LC Packings, Amsterdam, The Netherlands). After deconvolution of the averaged spectra, fractions containing synthetic chemokine with the correct M_r_ were folded by incubating them overnight at room temperature in folding buffer containing 1M guanidium chloride, 0.3mM reduced glutathione, 3mM oxidized glutathione and 3mM ethylenediaminetetraacetic acid (EDTA) in 150mM Tris (pH 8.6). Folded chemokines were purified in the same manner as the natural chemokines, using a C8 Aquapore RP-300 RP-HPLC column as described above, accompanied by on-line mass spectrometry to assure the correct molecular weight and purity of the synthetic proteins.

### Glycosaminoglycan and receptor binding properties

The ability of [3-NT^7^]CXCL12 to bind to glycosaminoglycans was compared to native CXCL12 using GAG binding plates (BD Biosciences, Franklin Lakes, New Jersey) coated overnight at room temperature with 25μg/ml heparin, heparan sulfate or dermatan sulfate (Iduron, Manchester, UK) in standard assay buffer (SAB; 100mM NaCl, 50mM sodium acetate, 0.2% (v/v) Tween-20; pH 7.2) as previously described [52]. Briefly, after thoroughly washing the plate with SAB buffer, the plates were blocked using SAB buffer enriched with 0.2% (w/v) gelatin for 1 hour at 37°C. After discarding the blocking buffer, serial dilutions of the synthetic CXCL12 variants in blocking buffer were added to the heparin-coated plates and incubated at 37°C for two hours. After a thorough washing step using SAB, bound chemokines were detected using the same procedure as described for the ELISA protocol. The data are shown as the percentage of maximum bound CXCL12, which was determined for each experiment.

The binding properties of the two different CXCL12 variants to CXCR4 and ACKR3 were measured using CXCL12^AF647^ (Almac, Craigavon, Northern Ireland) in a previously described competition assay [64]. CHO cells, transfected with CXCR4 or ACKR3, were treated for 20 seconds with EDTA-enriched trypsin (Lonza) to detach them from the culture flasks. Cells were resuspended in Ham's F12 medium enriched with 10% (v/v) FBS and were allowed to restore their receptor expression after trypsinization for two hours at room temperature. After a washing step, 1.5 × 10^6^ cells/ml were incubated for 1 hour on ice with 12.5nM CXCL12^AF647^ and varying concentrations of unlabeled CXCL12 variants in a final volume of 200μl RPMI 1640 enriched with 2% (v/v) FBS. After extensive washing in the same buffer, cells were fixed in RPMI 1640 with 2% (v/v) FBS and 0.4% (w/v) formaldehyde. The amount of fluorescence present on the cells was measured using flow cytometry (FACSCalibur flow cytometer, BD Biosciences).

### Receptor internalization

Internalization of CXCR4 was measured using the CHO-CXCR4 cell line. Cells were removed from their cell culture flasks and allowed to restore basal receptor expression as described above [64]. Hereafter, the cells were washed twice using RPMI 1640 with 0.5% (v/v) human serum albumin (HSA, Belgian Red Cross). Finally, the cells were resuspended in this medium to a final concentration of 5 × 10^6^ cells/ml. In a 96-well plate, 100μl of this suspension was added to each well. Different concentrations of the tested CXCL12 forms were added to these cell cultures and allowed to incubate for one hour at 37°C. Hereafter, the cells were put on ice for the remainder of the experiment to prevent further changes in the receptor expression level. Also 200μl of ice-cold assay buffer (phosphate-buffered saline (PBS) with 2% (v/v) FBS) was added to the cells as a first washing step. A specific CXCR4/CD184 (Clone 12G5; BD Biosciences) antibody was diluted in ice-cold assay buffer to a working concentration of 12.5μg/ml and allowed to incubate for 30 minutes. After two washing steps in ice-cold assay buffer, the cells were incubated for 30 minutes with 1.3μg/ml of phycoerythrin-labeled goat anti-mouse IgG (BD Biosciences) in ice-cold assay buffer. After two washing steps in ice-cold assay buffer the cells were fixed using assay buffer with 0.4% (w/v) formaldehyde. The CXCR4 expression on the cell surface was measured using flow cytometry (FACSCalibur flow cytometer). The data are presented as percentages of remaining receptor expression, where the maximal receptor expression was determined as the amount of fluorescence after stimulation with assay buffer only and incubation (37°C, 1 hour). Prevention of internalization and the absence of competition between the anti-CXCR4 antibody and CXCL12 was confirmed by keeping the cells on ice during the incubation using the highest dose of the CXCL12 forms (data not shown).

### Signal transduction assays

β-arrestin 2 recruitment to CXCR4 was performed using the PathHunter β-arrestin assay (DiscoveRx) as described previously [96], using C2C12 CXCR4 transfectants. CXCR4 was C-terminally linked with a ProLink tag (PK; DiscoveRx) which combines with the second fragment of β-galactosidase to which β-arrestin 2 is linked (enzyme acceptor (EA)-tagged β-arrestin 2, DiscoveRx). Upon recruitment, both fragments (PK and EA) combine to generate the active enzyme. The recruitment of β-arrestin 2 to CXCR4 can thus be quantified measuring the produced luminescence one hour after addition of the appropriate substrates from the kit. β-arrestin 2 recruitment to ACKR3 was performed in a similar manner using U2OS that are stably transfected with EA-tagged β-arrestin 2 and transiently transfected with PK-tagged ACKR3.

Calcium mobilization was measured using both THP-1 and CXCR4-transfected CHO cells as previously described [52]. Briefly, the cells were resuspended in their respective culture medium with the addition of 2.5μM of the calcium binding fluorescent dye Fura-2 (Molecular Probes, Invitrogen) and 0.01% (w/v) pluronic F-127 (Sigma-Aldrich) at a concentration of 10^7^ cells/ml. To the CHO-CXCR4 cells also 125μM probenicid was added to block the release of Fura-2 from the cell. The cells were loaded with Fura-2 during 30 minutes at 37°C. To account for the autofluorescence, 10^7^ cells were not treated with Fura-2. After washing the cells, they were resuspended in calcium buffer to a final concentration of 10^6^ cells/ml and kept at 4°C from this point onwards. Calcium buffer was made using Hanks' balanced salt solution (HBSS; Gibco) with 1mM Ca^2+^ and 10% (v/v) FBS, buffered with 10mM HEPES/NaOH (Sigma-Aldrich) to pH 7.4 for THP-1 cells and pH 7.0 for CHO-CXCR4 cells. Cells were shortly incubated at 37°C before measuring the calcium concentration after stimulation with various concentrations of the different CXCL12 forms. Fura-2 fluorescence was measured on a LS50B spectrofluorimeter (PerkinElmer) at 510nm after alternating excitation at 340nm and 380nm.

Phosphorylation of Akt was tested on CHO-CXCR4 cells and phosphorylation of ERK1/2 was tested using CHO-CXCR4 cells and fresh monocytes as described previously [97]. The cells were stimulated for 2 minutes with various concentrations of CXCL12 and [3-NT^7^]CXCL12. The amount of phosphorylated ERK1/2 and Akt was determined using specific ELISA assays for Phospho-ERK1(T202/Y204)/ERK2(T185/Y187) and Phospho-Akt (S473) (duosets; R&D Systems). The ratio of phosphorylated ERK1/2 or Akt and the total amount of protein was calculated for each sample. The data are depicted as percentages compared to unstimulated control.

The inositol turnover assay was performed using CXCR4-transfected COS-7 cells which were transiently transfected using the previously described calcium phosphate transfection method [98]. Both cDNA of CXCR4 and the chimeric G protein G*α*Δ6qi4myr (7TM-Pharma, Hørsholm, Denmark) were transfected to allow the cells to activate phospholipase C upon ligand stimulation. One day after transfection, cells were diluted to a concentration of 3.5 × 10^4^ cells/ml in a poly-D-lysine-coated 96-well plate. The cell medium was enriched with 0.5μCi of Myo-[2-^3^H(N)]-inositol and the cells were incubated overnight at 37°C in 10% CO_2_. The radioactive medium was removed and cells were washed twice using HBSS. Hereafter, cells were incubated for 90 minutes at 37°C with 100μl of 10mM LiCl in HBSS together with the CXCL12 forms in various concentrations. Subsequently, cells were lysed using 10mM formic acid with the plate put on ice for 30 minutes. Together with 35μl of cell extract, 80μl of 1:8 diluted YSi Poly-D-Lysine-coated beads (PerkinElmer) were added to a new 96-well plate and stirred vigorously. After centrifugation, the beads were allowed to react with the extract for at least 8 hours before luminescence was measured using the Packard Top Counter NXT^TM^ (PerkinElmer).

### Chemotaxis assays

Cell migration of THP-1 cells induced by the different CXCL12 variants was tested using a 96-well multiscreen plate (Millipore) as previously described [97]. Samples were diluted in RPMI 1640 with 0.1% (w/v) bovine serum albumin (BSA; Sigma-Aldrich). The THP-1 cells were diluted to a final concentration of 3.5 × 10^6^ cells/ml in RPMI without phenol red and L-glutamine (Lonza) and incubated for 3 hours at 37°C. Hereafter, the filter plate with 5μm pores was removed and the migrated cells were quantified using the ATPlite kit (PerkinElmer). Chemotactic indices were calculated by dividing the luminescence intensity of the test sample by the luminescence intensity of the cells that migrated towards chemotaxis buffer alone.

Lymphocyte chemotaxis was performed in Boyden microchambers using unfractionated PBMCs that were allowed to migrate towards different concentrations of the CXCL12 forms under conditions which favor migration of the lymphocyte fraction of the PBMCs. These conditions include a 4 hour incubation period, a PBMC concentration of 10 × 10^6^ cells/ml and the usage of a 5μm pore size, PVP-free fibronectin-coated (25μg/ml) polycarbonate membrane [99]. Migrated cells were fixed and colored with Hemacolor solutions (Merck, Darmstadt, Germany) and counted microscopically using immersion oil and a 500-fold magnification. Chemotactic indices were calculated by dividing the amount of migrated cells by the amount of cells migrated towards buffer (HBSS enriched with 0.5% (v/v) HSA) alone.

### *In vivo* cell migration assay

The effects of nitration of CXCL12 on the ability of CXCL12 forms to attract lymphocytes *in vivo* by extravasation from the blood circulation into the tibiofemoral articulation were examined by *intra articular* (i.a.) injection of the different CXCL12 forms in 8 weeks old wildtype C57BL/6 mice. Three days prior to the experiment, the water of the mice was changed to a 1.7mg/ml solution of the CD26 inhibitor sitagliptin [Merck Sharpe & Dohme (MSD), Whitehouse Station, NJ)] in water to partially block the activity of this enzyme for which CXCL12 is an efficiently cleaved and inactivated substrate [57]. Endotoxin levels in the injected samples were tested with the *Limulus* amoebocyte lysate test (Cambrex, East Rutherford, NJ) and were lower than 0.125 pg LPS per μg chemokine. Different concentrations of the chemokine forms, diluted in a 0.9% (w/v) NaCl solution were injected i.a. as described previously [100], mice were placed under anaesthesia using 3.75% (w/v) ketamine, 0.25% (w/v) xylazine in PBS and injected i.a. with 10μl of the chemokine dilutions. After 3 hours of incubation, the mice were sacrificed and the articular cavity was washed using 3% (w/v) BSA in PBS. Total leukocytes were counted using a Neubauer chamber and Turk's staining solution. Hereafter, the samples were counted differentially on May-Grünwald-Giemsa stained Cytospins (Shandon III). Experiments were performed in the animalia of the University of Minas Gerais and Leuven. All experiments using laboratory animals were reviewed and approved by the Animal Ethical Committee of the University of Minas Gerais and the Animal Ethical Committee of the University of Leuven.
